# Wellbeing Among Police Personnel Investigating Online Child Sexual Abuse: A Scoping Review

**DOI:** 10.1177/15248380251358228

**Published:** 2025-08-20

**Authors:** Robert Lundmark, Alva Lindholm, Ola Lindberg, Oscar Rantatalo

**Affiliations:** 1 Department of Psychology, Umeå University, Sweden; 2 Department of Health, Education, and Technology, Luleå University of Technology, Sweden; 3 Department of Learning, Umeå University, Sweden

**Keywords:** child sexual abuse, digital policing, secondary trauma, stress, coping

## Abstract

Online child sexual abuse (CSA) crimes have increased significantly in recent years, reflecting broader access to the internet and the global proliferation of CSA content. In response, national police forces have established specialized investigative teams. Notably, repeated exposure to CSA material has been identified as a significant stressor for police personnel, potentially increasing the risk of secondary traumatic stress and burnout. Therefore, understanding and addressing the impact of continuous CSA exposure on personnel’s wellbeing is essential within this area of digital policing. This scoping review aims to synthesize and report the existing empirical research on the wellbeing of police personnel involved in online CSA investigations. Following established guidelines, we searched four electronic databases—Scopus, Web of Science (Clarivate), PsycINFO, and SocIndex—for articles published between 2000 and 2024. We also conducted reference mining of the included studies. In total, 33 articles met the inclusion criteria: empirical studies published in English, in peer-reviewed journals, and focused on the wellbeing of personnel investigating online CSA. Findings reveal substantial variation in reported wellbeing. Individual coping strategies, as well as organizational resources and support, play a critical role in how personnel manage the demands of this work. Based on these insights, we recommend that online CSA police units implement clear and proactive strategies to safeguard personnel’s wellbeing.

## Introduction

With the increased access to the internet and social media, online child sexual abuse (online CSA) has increased significantly over the last two decades (Ali et al., 2023). Today, the internet serves as a central platform for sexual abuse activities, providing offenders with opportunities to locate victims and to share CSA images and videos (Kloess et al., 2014). In response, national police forces have developed specialized units to investigate online CSA crime. These units consist of dedicated personnel, including lead investigators, investigators, digital forensic specialists, and open-source intelligence analysts (Lindholm et al., 2024). Although policing strategies may vary due to differences in national legislation and priorities, a shared goal among these units is to identify victims and prosecute offenders. Achieving this goal requires personnel to regularly view, review, and assess CSA material as part of their daily work tasks (Spencer et al., 2021).

Unsurprisingly, continuous exposure to CSA material has been identified as a major stressor that can severely impair police personnel’s wellbeing (Krause, 2009). Investigations often involve prolonged viewing and listening to graphic and disturbing content, a process that many would consider traumatizing (Spencer et al., 2021). This burden is compounded by the growing number of cases, where successful investigations may not only stop ongoing abuse but can also save children’s lives (Lindholm et al., 2024). Additional stressors include the pressure to keep up with rapidly evolving technologies and the challenge of working within outdated legal frameworks (Cullen et al., 2020). Moreover, online CSA police personnel risk stigma by association with the crimes they investigate, and others (e.g., police personnel from other units) may distance themselves out of fear or contamination, leading to social isolation (Spencer et al., 2021).

As online CSA policing is a relatively recent phenomenon evolving alongside digitalization, research on the wellbeing of personnel handling these crimes is still emerging. Unlike traditional CSA investigations, which seldom require personnel to be visually or auditorily exposed to abuse, members of online CSA units are routinely subjected to such exposure (Krause, 2009). Accordingly, the unique nature of the work renders this field exceptional in the types of stressors personnel encounter (Jewkes & Andrews, 2005). Studying the challenges of online CSA police work, specifically, including the work environment and wellbeing of the personnel working to solve these crimes, is hence an important task for researchers (Franqueira et al., 2018). This is particularly crucial for tailoring support systems and designing interventions to prevent declines in their mental health status and accompanying high turnover rates among these highly specialized employees (Burns et al., 2008).

Given the novelty of the crime type, research on this specific population has been sparse, fragmented across fields such as policing, psychology, and traumatology, and lacking systematic synthesis. In the present study, we aim to address this issue by charting and summarizing existing empirical work on the wellbeing of police personnel working with online CSA investigations. Our findings will clarify the current state of knowledge, identify gaps in the literature, and provide a foundation for future research. In addition, they can offer valuable insights for police organizations seeking to better support the wellbeing of personnel working in this highly demanding area.

## Methods

Research on the wellbeing of police personnel involved in online CSA investigations is still emerging, and the primary need at this stage is to provide an overview of existing research and findings. In line with this objective, we employed a scoping review methodology, as recommended by Munn et al. (2018), to map the current body of literature. The process followed the five stages outlined by Arksey and O’Malley (2005), with clarifications by Levac et al. (2010): (a) identify the research question; (b) identify relevant studies; (c) study selection; (d) chart the data; and (e) collate, summarize, and report results. In addition, we used the PRISMA-ScR checklist (Tricco et al., 2018) to guide the reporting of our findings.

### Research Questions

Based on the aim of this paper, the following research questions were specified to guide the review process:

1. What is known about the wellbeing of police personnel working with online CSA investigations?
2. How does exposure to CSA material and the use of different coping strategies influence the wellbeing of these personnel?
3. What intra-individual and organizational barriers and resources for wellbeing have been identified?
4. How can the wellbeing of online CSA police personnel be maintained and/or improved?

### Identification of Relevant Studies

To examine potential associations between exposure to online CSA work tasks and wellbeing outcomes among police personnel, we based our inclusion criteria on the Population, Exposure, and Outcome framework (Moola et al., 2015). Accordingly, the included population was restricted to police personnel involved in online CSA investigations, with wellbeing as the outcome of interest. Following Sonnentag et al.’s (2023) recommendations, we adopted a broad definition of wellbeing, encompassing personnel’s evaluations and perceptions of their overall situation, beyond a narrow focus on merely physical health (e.g., musculoskeletal symptoms). This includes both hedonic wellbeing (i.e., subjective experience of pleasure and positive affect), as well as eudaimonic wellbeing (experience of meaning, pursuit of goals in alignment with values, etc.; Ryan & Deci, 2001). Furthermore, evaluation of these potential physical and psychological wellbeing states and conditions contains both “positive” (e.g., satisfaction, vigor) and “negative” (e.g., stress, burnout) components, reflecting a range of wellbeing experiences (Sonnentag et al., 2023).

Based on our research questions, we included only empirical studies using primary data, either quantitative or qualitative in design. Eligible studies were required to be published in English in international peer-reviewed journals with a registered impact factor (of any level) or to score at least 1 on the Norwegian Register for Scientific Journals, Series and Publishers (Sivertsen, 2018), to ensure rigor in reporting. Given the evolution of internet use over the past two decades, only studies published between 2000 and 2024 were considered.

With assistance from the university library at, we developed a search strategy. In this iterative process, multiple search strings were constructed based on 11 identified “gold standard” articles. Each database search was followed by a screening of titles to refine the strategy and optimize the balance between sensitivity and specificity. Ultimately, search strings were constructed using only the Population (P) and Exposure (E) elements, allowing wellbeing outcomes (O) to be evaluated more carefully during the study selection phase. The primary searches were conducted in September 2024 across four electronic databases: Scopus, Web of Science (Clarivate), PsycINFO, and SocINDEX. Complete search strategies for each database are provided in Appendix 1.

### Study Selection and Data Charting

Titles and abstracts identified through database searches were imported into the software Rayyan (Ouzzani et al., 2016), where duplicates were removed. Subsequently, the first and second authors (RL and AL) independently screened all articles. To ensure inter-rater reliability, the third and fourth authors (OL and OR) independently reviewed a random selection of 10 articles. Weekly meetings were held during the screening process, where the third and fourth authors helped resolve any discrepancies or uncertainties between the first and second authors. Articles where disagreement remained after discussion were included for full-text screening.

All included articles were downloaded and reviewed in full text by at least two authors. Any disagreements were addressed in weekly meetings to reach a consensus. Relevant data were then systematically charted in Excel, including bibliographic details, study population, study design, theoretical framework, methodology, main findings, and proposed implications. As a final step, reference lists of included articles were screened to identify potentially overlooked studies. All four authors participated in the data extraction and charting process, which was continuously discussed in weekly meetings until consensus was achieved.

### Collating, Summarizing, and Reporting Results

From the charted data, we extracted information to describe the included studies and their populations, and to answer each research question. For each question, a separate worksheet was created in Excel. When more extensive information was available, the extracted information was coded and categorized based on commonalities. This process followed three phases—preparation, organization, and reporting—inspired by Elo and Kyngäs’ (2008) approach to inductive qualitative content analysis. The first and second authors led this process, with regular consultations from the third and fourth authors to ensure consistency in the synthesis and presentation of results.

## Results

The database searches yielded a total of 1,086 articles. After removal of duplicates, 739 unique records remained and were screened at the title and abstract level. Following the blinded screening process in Rayyan, 36 articles were retained for full-text assessment. During this stage, eight articles were excluded: two for not including primary data, three for not focusing on personnel involved in online CSA investigations, and three for not addressing wellbeing as an outcome. After full-text screening, 28 articles met the inclusion criteria. An additional five relevant articles were identified through reference list screening of the included studies. In total, 33 articles were deemed eligible and included in the final review.

**Figure 1.**
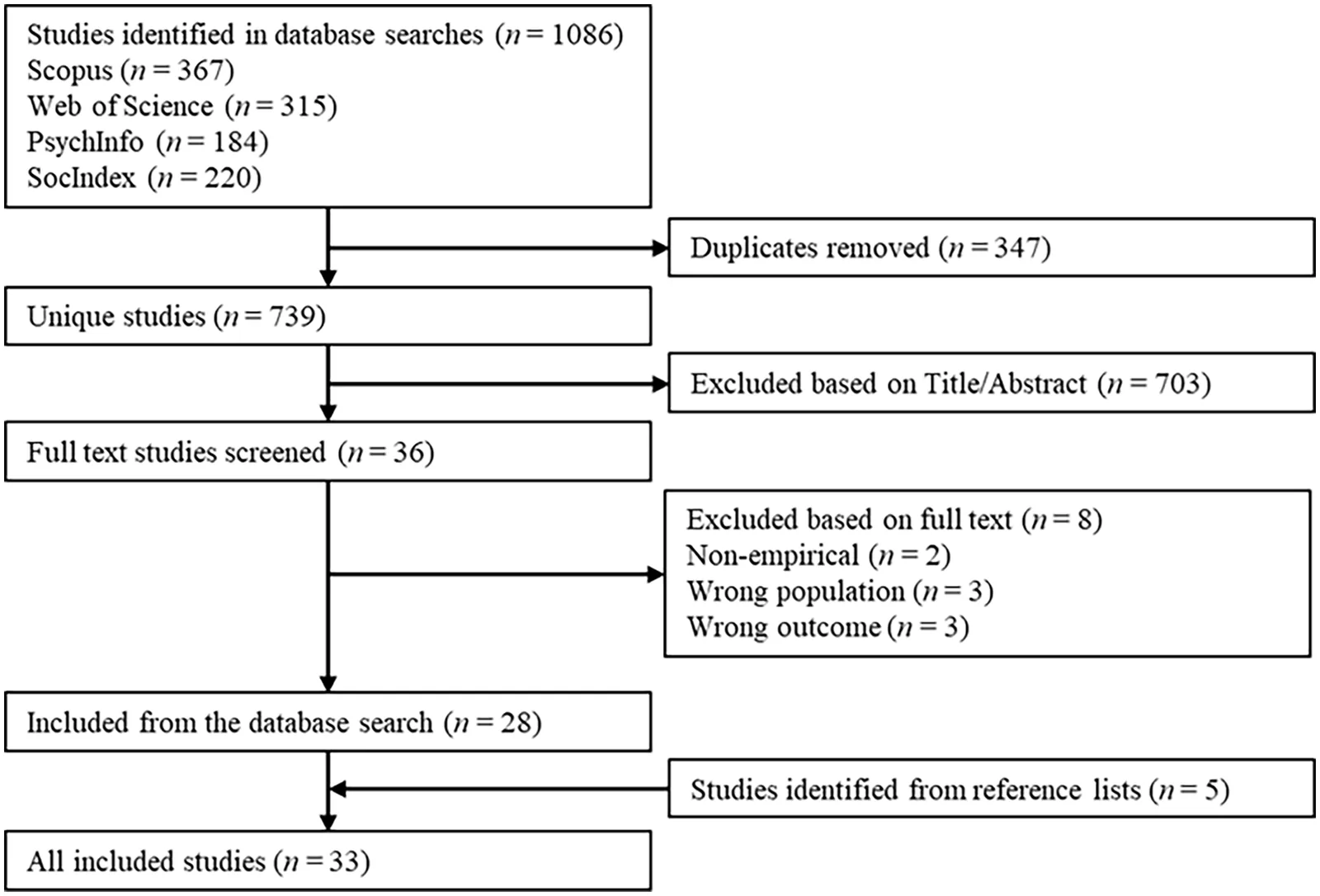
PRISMA flowchart of the review process.

The review process, including inclusion and exclusion at each stage, is illustrated in Figure 1 (PRISMA flowchart).

### Study Characteristics

**Figure 2.**
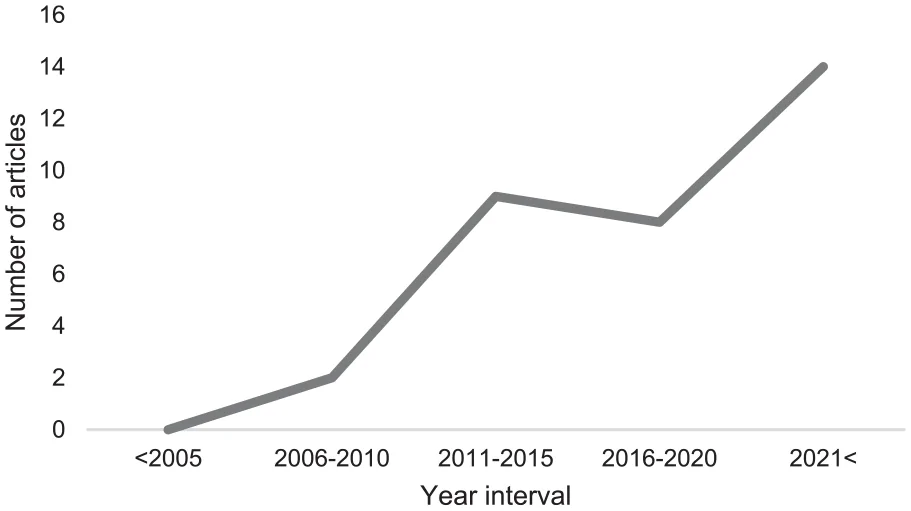
Number of included studies from 2000 to 2024 in 5-year intervals. *Note.* The included studies were published from 2008 to 2024.

A large part of the studies (*n* = 14) was conducted in the last 4 years (i.e., 14 studies since 2021, see also Figure 2), potentially reflecting both a rise in this type of crime and increased scholarly attention to the challenges associated with online CSA investigations.

A detailed description of the study characteristics of the included articles is presented in Table 1. Out of the 33 included articles, most were based on data from the United States (*n* = 14). The United Kingdom (*n* = 8), Australia (*n* = 5), and Canada (*n* = 2) were other countries of origin. One article was based on data from both the United States and the United Kingdom, and three articles included data from a multitude of countries. In total, 24 of the articles involved a mixed population (e.g., including both police officers and digital forensic examiners), whereas 5 focused only on investigators, and 4 focused only on digital forensic examiners. In terms of design, 20 of the articles used a cross-sectional survey design, 10 used interviews, and 2 were based on a mixed-method design (i.e., 1 using interviews and observations and 1 using interviews and cross-sectional survey data).

**Table 1.** Study Characteristics.

Article	Country	Population	Study design
Bourke and Craun (2014a)	United States	Mixed: ICAC personnel (*n* = 600)^a^	Cross-sectional survey
Bourke and Craun (2014b)	United Kingdom & United States	Mixed: ICAC personnel (*n* = 600)^a^ + ICAC investigators (UK) (*n* = 288)	Cross-sectional survey
Brady (2017)	United States	Mixed: ICAC personnel (*n* = 433)	Cross-sectional survey
Burns et al. (2008)	Canada	Mixed: Royal Canadian Mounted Police integrated ICE team (*n* = 14)	Interviews
Burruss et al. (2018)	United States	Forensics: Digital forensics (*n* = 360)	Cross-sectional survey
Craun and Bourke (2014)	United States	Mixed: ICAC personnel (*n* = 508)	Cross-sectional survey
Craun and Bourke (2015)	United States	Mixed: ICAC personnel (*n* = 350)	Cross-sectional survey
Craun et al. (2015)	United States	Mixed: ICAC personnel (*n* = 600)^a^	Cross-sectional survey
Denk-Florea et al. (2020)	United Kingdom	Mixed: Digital forensic analysts (59 %), manager, investigator, supervisor and detective with current or previous experience of CSA (*n* = 22)	Interviews
Doyle et al. (2023)	United Kingdom	Mixed: ICAT—Police officers or any employee in contact with online CSA images (*n* = 6)^b^	Interviews
Fortune et al. (2018)	Multinational*	Mixed: Examiner, child abuse investigator, computer forensic examiner, investigator, and digital crime investigator/trainer (*n* = 5)	Interviews
Gewirtz-Meydan et al. (2023)	United States	Mixed: ICAC personnel (*n* = 500)^c^	Cross-sectional survey
Gewirtz-Meydan et al. (2024)	United States	Mixed: ICAC personnel (*n* = 470)^c^	Cross-sectional survey
Gullon-Scott and Johnson (2024)	United Kingdom	Forensics: Digital forensics (*n* = 114)	Cross-sectional survey
Leclerc et al. (2022)	Australia	Investigators: CSA online investigators (*n* = 29)	Interviews
Mitchell et al. (2022)	United States	Mixed: ICAC commanders (*n* = 54) investigators (*n* = 155)^c^	Interviews and cross-sectional survey
Mitchell et al. (2023)	United States	Mixed: ICAC personnel (*n* = 500)^c^	Cross-sectional survey
Mitchell et al. (2024)	United States	Mixed: ICAC personnel (*n* = 500)^c^	Cross-sectional survey
O’Brien et al. (2024)	United States	Mixed: ICAC personnel (*n* = 500)^c^	Cross-sectional survey
Perez et al. (2010)	United States	Investigators: Investigators at a federal law enforcement agency (*n* = 28)	Cross-sectional survey
Powell et al. (2014a)	Australia	Investigators: ICE investigators (*n* = 32)^d^	Interviews
Powell et al. (2014b)	Australia	Investigators: ICE investigators (*n* = 32)^d^	Interviews
Powell, et al. (2015)	Australia	Investigators: ICE investigators (*n* = 32)^d^	Interviews
Redmond et al. (2023)	United Kingdom	Mixed: Police officers working in CSA investigations (*n* = 661)	Cross-sectional survey
Seigfried-Spellar (2018)	United States	Mixed: ICAC personnel (*n* = 129)	Cross-sectional survey
Simonovska et al. (2023)	**Multinational	Mixed: personnel who at the time of the study were working or had worked in an online CSA unit, Mixed (*n* = 642)	Cross-sectional survey
Spencer et al. (2020)	Canada	Mixed: 70 interviews and two focus groups were conducted with Sex crime units’ personnel (adults, children, cyber/online; *n* = 70).	Interviews
Stewart and Witte (2020)	United States	Mixed: ICAC personnel with children 5–18 (*n* = 212)	Cross-sectional survey
Strickland et al. (2023)	United Kingdom	Forensics: Digital forensics analysts (*n* = 7)	Interviews
Tapson et al. (2022)	United Kingdom	Mixed: ICAT—Police officers or any employee in contact with online CSA images (*n* = 6)^b^	Interviews
Tehrani (2016)	United Kingdom	Mixed: ICAI (*n* = 126)	Cross-sectional survey
Tomyn et al. (2015)	Australia	Mixed: ICE investigators (*n* = 139) + non-ICE investigators (*n* = 102) + Mainstream sample (*n* = 55 697)	Cross-sectional survey
Wilson-Kovacs et al. (2022)	United Kingdom	Forensics: Four DFUs, 270 h of observation, Digital forensics (*n* = 67)	Observations Interviews

*Note.* *European police forces, United States, Canada, and international policing organization. **USA, Canada, Australia, UK, Switzerland, United Arab Emirates, Colombia, Denmark, Germany, France, “other.”

a, b, c, d Studies conducted on the same dataset.

CSA = child sexual abuse, DFU = Digital forensic units, ICAC = Internet crimes against children task force, ICAI = Internet child abuse investigators, ICE = Child Exploitation Investigators, ICAT = Internet Child Abuse Team.

### The Wellbeing of Online CSA Police Personnel

A range of different wellbeing outcomes were studied across the different studies (see Table 2). The most frequently studied wellbeing outcome in the included studies using a survey design was secondary traumatic stress (STS) or post-traumatic stress (PTS), with sexual PTS being the focus of one of these studies (Gewirtz-Meydan et al., 2023). STS are symptoms identical to PTS (i.e., intrusion, avoidance, and arousal) but derived from indirectly experiencing someone else’s trauma (Bride et al., 2004). The occurrence of burnout, anxiety, depression, and general/overall wellbeing was also studied to some degree. Turnover intentions and parenting/protectiveness behaviors were included in two separate studies as more distal outcomes. Positive outcomes in terms of different aspects of satisfaction (i.e., compassion, life-, or job-satisfaction) were less frequently studied.

**Table 2.** Wellbeing Outcomes Measured in the Included Survey Studies.

Wellbeing Measure	Article
Secondary/Post-traumatic stress (*n* = 15)	Bourke and Craun (2014a), Bourke and Craun (2014b), Brady (2017), Burruss et al. (2018), Craun and Bourke (2014, 2015), Craun et al. (2015), Gewirtz-Meydan et al. (2023, 2024), Gullon-Scott and Johnson (2024), Mitchell et al. (2023, 2024), Perez et al. (2010), Seigfried-Spellar (2018), Stewart and Witte (2020), Tehrani (2016)
Burnout (*n* = 5)	Brady (2017), Gewirtz-Meydan et al. (2024), Mitchell et al. (2024), Perez et al. (2010), Tehrani (2016)
Anxiety (*n* = 5)	Gewirtz-Meydan et al. (2023, 2024), Mitchell et al. (2023, 2024), Tehrani (2016)
Depression (*n* = 5)	Gewirtz-Meydan et al. (2023, 2024), Mitchell et al. (2023, 2024), Tehrani (2016)
*General/overall wellbeing (*n* = 5)	Mitchell et al. (2023, 2024), Seigfried-Spellar (2018), Simonovska et al. (2023), Tomyn et al. (2015)
Turnover intentions (*n* = 2)	Bourke and Craun (2014a), Perez et al. (2010)
Parenting/Protectiveness (*n* = 2)	Bourke and Craun (2014a), Stewart and Witte (2020)
Job-satisfaction (*n* = 2)	Bourke and Craun (2014a), Seigfried-Spellar (2018)
Compassion satisfaction (*n* = 1)	Brady (2017)
Life-satisfaction (*n* = 1)	Tomyn et al. (2015)

Note. *Measured either using a one-item question or using a multitude of indicators.

Overall, the results reflect that most online CSA police personnel do not suffer from impaired wellbeing (at least not on a clinical level), although many studies also indicate in-group variations. For the majority of study participants, symptoms of STS/PTS, burnout, depression, and anxiety seem to be on a low level, whereas positive wellbeing outcomes are on a high level. However, results of these studies also imply that for some, approximately 20% to 30 % (depending on sample), the symptoms of negative wellbeing are on a high or severe level (e.g., Bourke & Craun, 2014a; Brady, 2017). As expected, higher levels of STS were also positively associated with turnover intentions (Bourke & Craun, 2014a; Perez et al., 2010) and controlling parental behaviors (Bourke & Craun, 2014a; Stewart & Witte, 2020). Similar findings were also present in one of the more recent survey studies that reported on a variety of wellbeing indicators, where symptoms such as negative worldview, heightened protectiveness, and sleep disturbances were among the most frequently reported (Simonovska et al., 2023).

When compared to other populations, results are also somewhat inconclusive. For example, Perez et al. (2010) conclude that the level of burnout and STS is high compared with similar professions (i.e., social workers and forensic interviewers of child abuse victims). In contrast, Tomyn et al. (2015) found that the wellbeing of online CSA police personnel does not differ substantially from other police personnel and that it is higher than the Australian adult population at large. Similarly, Tehrani (2016) concluded that the mean levels of anxiety and depression found among online CSA police personnel were below levels found in other occupations.

These results are reflected in the interview studies, where reported wellbeing in general is high, but not for all (e.g., Fortune et al., 2018). For example, in 1 study, 30 out of 32 interviewed online CSA police personnel reported that their work did not have a substantial negative impact on their wellbeing and that they would recommend the work to others (Powell et al., 2014a). The results also indicate that, for most individuals, stress levels decrease over time because of habituation (Denk-Florea et al., 2020; Fortune at al., 2018).

However, reports on negative experiences in the form of flashbacks, intrusive thoughts, mood swings, disturbed sleep, hypervigilance, helplessness, and sadness indicate that the job also involves distressful events (Denk-Florea et al., 2020; Powell et al., 2015; Strickland et al., 2023). Furthermore, spill-over effects on their personal life in terms of distrust of others, overprotectiveness of children, and cynicism toward others and the world in general were also recurring themes in the interview studies (Denk-Florea et al., 2020; Powell et al., 2015; Strickland et al., 2023; Spencer et al., 2020; Tapson et al., 2022).

### Factors Influencing the Wellbeing of Online CSA Police Personnel

Given the substantial variation in the level of wellbeing of online CSA police personnel, several of the articles strive to identify how different factors can explain the discrepancies. We have categorized these factors in terms of the degree of exposure and the type of online CSA content, individual characteristics and coping strategies, as well as leader support, conditions in the workgroup, and conditions in the organization and overall system.

### Exposure and Type of Online CSA Content

From reviewing the articles, the associations between frequency, duration, and content of CSA material with the wellbeing of online CSA police personnel are ambiguous. Some report that the amount and frequency of exposure play a major role (Bourke & Craun, 2014a; Burns et al., 2008; Burruss et al., 2018, Perez et al., 2010). Others conclude that frequency and duration of exposure are not systematically related to wellbeing (Gewirtz-Meydan et al., 2023; Mitchell et al., 2023). It remains unclear whether desensitization to the material over time constitutes a protective mechanism or a maladaptive emotional detachment strategy. It could be seen as an efficient mechanism allowing for more productive work, but it could also be seen as a less beneficial emotional detachment strategy that affects wellbeing negatively over time (Powell et al., 2015).

Nevertheless, the form and content of the material seem to be important for its detrimental effects. For example, video with audio is reported to be more distressful than pictures (Powell et al., 2015). Material with violent content (Mitchell et al., 2023), high intensity (Burns et al., 2008), material with infants, and parents abusing their children (Powell et al., 2015), was also considered to be extra distressing.

### Individual Characteristics and Coping Strategies

In some of the articles, gender differences are examined. All of these points toward women experience somewhat more adverse effects than men when working in online CSA units (Bourke & Craun, 2014a; Gewirtz-Meydan et al., 2023; Gullon-Scott & Johnson, 2024; Tehrani, 2016). Whether these differences are related to the work tasks or a reflection of other factors (e.g., women’s wellbeing at work being generally lower than men’s) were not clarified. Furthermore, neuroticism was in one study associated with high levels of anxiety, depression, burnout, and STS/PTS (Tehrani, 2016). Doyle et al. (2023) found that personnel with prior trauma or with children of their own were at particular risk for adverse wellbeing outcomes. Among those who are parents, discomfort in expressing intimacy with one’s own children was found to be more pronounced among men than women (Craun et al., 2015).

Conversely, Mitchell et al. (2023) found that being married increased chances of beneficial wellbeing outcomes. Online CSA police personnel whose appraisal of the challenges at work is cognitive-based and system-focused (e.g., frustrated with the workload), compared to personnel whose appraisal is emotion-based and victim-focused (e.g., wanted to do more for the victim), have also been suggested to enjoy a higher overall wellbeing (O’Brien et al., 2024).

Difficulties associated with viewing CSA material have been found to be a major predictor of hampered wellbeing among online CSA police personnel (Perez et al., 2010; Gullon-Scott & Johnson, 2024). Therefore, identifying strategies that can help alleviate stress from repeated exposure to CSA pictures and videos is of vital importance. Although results from one study suggest that coping strategies do not contribute significantly to this line of work (Gullon-Scott & Johnson, 2024), several other studies highlight the importance of different coping strategies for explaining how stress can be mitigated. Overall, these coping strategies focus either on the task of viewing material to make it less intrusive, or on ways for decompressing from work (see Table 3 for a compilation of mentioned coping strategies).

**Table 3.** A compilation of the Mentioned Coping Strategies.

Strategies for viewing	How it’s done
Mental preparations/focus	Preparation routines before watching, determining when, where, and how to view (Burns et al., 2008; Mitchell et al., 2023), taking an analytical viewpoint, shutting down emotions (Denk-Florea et al., 2020; Fortune et al., 2018), focus on the task and what can be achieved (Fortune et al., 2018), focus on evidence and remaining analytical (Burns et al., 2008).
Limiting the time spent watching	Only watching short sequences, taking regular breaks (Burns et al., 2008; Denk-Florea et al., 2020; Fortune et al., 2018; Mitchell et al., 2023; Wilson-Kovacs et al., 2022).
Manipulate auditive/visual input	Turning audio down or off when watching (Denk-Florea et al., 2020; Mitchell et al., 2023; Wilson-Kovacs et al., 2022), flipping images to another view (e.g., smaller window; Denk-Florea et al., 2020), narrowing the focus to relevant factors (Mitchell et al., 2023), and listening to music while working (Fortune et al., 2018; Mitchell et al.,2023; Wilson-Kovacs et al., 2022)
Strategies to decompress	How it’s done
Detaching from work	Utilizing commuting time to change mode (Strickland et al., 2023), hobbies and exercise for maintaining a healthy lifestyle (Fortune et al., 2018; Strickland et al., 2023), maintaining boundaries between work and home (Fortune et al., 2018; Powell et al., 2014a), upholding a stable home environment (Craun et al., 2015; Strickland et al., 2023)
Talking/seeking support	Talking to others (e.g., colleagues, spouses; Denk-Florea et al., 2020; Fortune et al., 2018; Mitchell et al., 2023; Wilson-Kovacs et al., 2022), utilizing psychological support (Fortune et al., 2018)
Humor	Using lighthearted humor (Craun & Bourke, 2014), using “dark” humor (Denk-Florea et al., 2020)
(Re-)appraisal	Entertaining pleasant thoughts (Denk-Florea et al., 2020: Mitchell et al., 2023), reminding oneself of the importance of the work (Doyle et al., 2023; Mitchell et al.,2023)
Variating tasks	Day-to-day variation of workload (Fortune et al., 2018), taking control over assigning work (Gewirtz-Meydan et al., 2023, 2024; Mitchell et al., 2023).

*Note.* The presented coping strategies are derived from both survey and interview studies. Examined strategies that were not statistically significant related to wellbeing outcomes in quantitative analysis are not represented.

In addition to coping strategies that can help mitigate stress, several articles also mention maladaptive strategies, as well as warning signs of hampered wellbeing. The use of alcohol after work (Craun & Bourke, 2015; Fortune et al., 2018; Mitchell et al., 2023), sugar consumption (i.e., snacks or drinks; Denk-Florea et al., 2020), and increased tobacco use during the last year (Bourke & Craun, 2014a) have all been found to indicate deterioration of wellbeing among online CSA police personnel.

Furthermore, avoidance of specific activities and ignoring the harm (Denk-Florea et al., 2020), withdrawal (Powell et al., 2014a), and using denial (Bourke & Craun, 2014a) are also mentioned as less favorable strategies. The use of humor seems to be a double-edged sword, as some forms are less beneficial ways of coping with stress. Using humor at the expense of victims (Craun & Bourke, 2015), as well as using gallows humor (Craun & Bourke, 2014), can be seen as warning signs. Display of negative affect (e.g., irritability or teariness) or changes in physical appearance (e.g., weight loss/gain or looking fatigued) were other observed warning signs (Powell et al., 2014a).

### Intra-Individual and Organizational Barriers and Resources for Wellbeing

Beyond exposure to online CSA material and the use of coping mechanisms, a few of the studies focused on the overall work environment to identify barriers and resources for wellbeing. High work pressure paired with insufficient resources—leading to long work hours were repeated themes in these studies (Powell et al., 2014b; Simonovska et al., 2023). The studies identify common barriers such as high workloads, understaffing, inadequate IT tools, time constraints, unsuitable physical work environments, and a workplace culture that either stigmatizes seeking psychological support or lacks access to it (Mitchell et al., 2022; Powell et al., 2014b; Redmond et al., 2023; Simonovska et al., 2023; Tomyn et al., 2015).

Conversely, resources and facilitators for wellbeing were found mainly in the close bond that developed over time in the work groups and units, providing both task-related and emotional support (Denk-Florea et al., 2020; Simonovska et al., 2023; Tomyn et al., 2015). The results of the investigations, when successful, were also a major contributor to wellbeing, as were support from managers as well as from family and friends (Denk-Florea et al., 2020; Simonovska et al., 2023). Furthermore, the availability of psychological support was seen as a relief to the experienced strain, and a complement to the support given by colleagues and others (Denk-Florea et al., 2020).

Concerning the physical work environment, the possibility to access separate rooms or spaces was emphasized (Denk-Florea et al., 2020; Simonovska et al., 2023) but also seen as a risk since it could reduce supportive interaction and increase isolation (Powell et al., 2014b). Established work routines such as time limits for exposure to distressing material, as well as flexibility in work-task rotation, were additionally proposed to facilitate stress-resistance as it reduced lengthy exposure to online CSA material (Denk-Florea et al., 2020).

### Maintaining and/or Improving Online CSA Police Personnel Wellbeing

Although none of the included studies were interventions, almost all gave extensive suggestions for how wellbeing could be upheld and/or improved in online CSA police units. These suggestions included several different themes, defining both preventive and promotive measures, and are based on findings from different police units and forces, in different countries and at different times. However, many of the suggestions are recurrent and, therefore, could be interpreted as potentially operative across settings.

We have categorized the recommendations in two overarching dimensions: workplace prerequisites and screening and intervening (see Table 4). These dimensions each include six categories of suggested measures that online CSA police organizations can take to improve the wellbeing of their personnel. *Workplace prerequisites* embrace aspects of culture, training, staffing, work routines, technology, and the design of the physical workplace. All of which are highlighted in the articles as fundamentals for creating resilient workplaces. *Screening and intervening* refer to the aspects of monitoring, stigma reduction, psychological support, debriefing, family support, and wellness programs. These mainly focus on identification and acting upon signs of hampered wellbeing. The two dimensions and their different categories of measures can be seen as interlinked. For example, training to enhance the use of beneficial coping strategies could also be seen as part of a wellness program, or efforts to reduce stigma as a way of enhancing a caring culture at the workplace. Thus, our categorization of these measures is merely a way of collating all suggestions.

**Table 4.** Suggestions for Upholding and Improving Wellbeing in Online CSA Police Organizations.

Workplace prerequisites	
*Culture*	Articles
By addressing the organization’s culture, it is suggested that multiple aspects that play a role for the wellbeing of online CSA personnel can be tackled. Building a culture in which wellbeing is prioritized and highly valued, creating a supportive community spirit with strong team cohesion and collaboration with fast response to intrateam conflicts is advocated in these proposals.	Bourke and Craun (2014b), Denk-Florea et al. (2020), Redmond et al. (2023), Strickland et al. (2023), Tomyn et al. (2015), Wilson-Kovacs et al. (2022)
*Training*	
Educating personnel, managers, families and friends, and mental health professionals to raise awareness about CSA investigative work, common reactions, and ways to promote healthy coping may provide beneficial strategies to handle strain and for drawing support from personnel’s wider network. Training can also involve parenting or relational instructions and advice to reduce the chances of spill-off reactions	Brady (2017), Burns et al. (2008), Burruss et al. (2018), Craun and Bourke (2014), Fortune et al. (2018), Gewirtz-Meydan et al. (2023), Gullon-Scott and Johnson (2024), Mitchell et al. (2023), Perez et al. (2010), Simonovska et al. (2023), Stewart and Witte (2020)
*Staffing*	
Staffing involves allocating sufficient financial and human resources to develop appropriately sized teams to reduce the overall workload. It also includes developing adequate processes for human resource management, such as for selecting and introducing team members and leaders	Burns et al. (2008), Fortune et al. (2018), Powell et al. (2014a, 2014b), Simonovska et al. (2023), Tapson et al. (2022), Tomyn et al. (2015)
*Work routines*	
Policies and procedures to enable functional and flexible routines are argued to be a central prerequisite for personnel wellbeing. This encompasses giving them control over the work, self-regulation policies for the time and frequency of interaction with CSA material, limits for how many cases they should manage, and the duration of service in a specific role. It can also include procedures to make sure that all involved are appraised of the final case solution and sharing of experiences for collective learning	Brady (2017), Bourke and Craun (2014b), Mitchell et al. (2023), Perez et al. (2010), Tomyn et al. (2015), Tapson et al. (2022)
*Technology*	
Keeping up-to-date and providing adequate technical equipment as well as software is vital for processing cases efficiently and for reducing unnecessary exposure to CSA material.	Burruss et al. (2018), Fortune et al. (2018), Powell et al. (2014b), Simonovska et al. (2023)
*Physical workplace*	
Optimizing the physical workplace to fit with the unique tasks of online CSA investigations is recommended as a measure to increase possibilities for the integrity of the victims, focused attention when viewing, and at the same time promote opportunities for team scaffolding	Denk-Florea et al. (2020), Powell et al. (2014b), Simonovska et al. (2023)
Screening and intervening	
*Monitoring*	Articles
To detect signs of hampered wellbeing, screening for signs of stress and burnout is vital. For example, through annual assessments with standardized tools and/or through dialog between coworkers, or through team leader monitoring. Such screening can also provide information for more targeted interventions on different levels (i.e., individual, group, and organizational level)	Burns et al. (2008), Burruss et al. (2018), Doyle et al. (2023), Gullon-Scott and Johnson (2024), Mitchell et al. (2024), O’Brien et al. (2024), Powell et al. (2014a), Tapson et al. (2022), Tehrani (2016), Wilson-Kovacs et al. (2022)
*Stigma reduction*	
Stigma reduction refers to working for recognition and awareness to gain respect from others and reduce fear of contamination though association. It also refers to reducing stigma related to help seeking behaviors among online CSA personnel when struggling with mental health or exhaustion from work.	Brady (2017), Fortune et al. (2018), Gullon-Scott and Johnson (2024), Mitchell et al. (2023), Redmond et al. (2023), Spencer et al. (2020)
*Psychological support*	
Access to support from professionals in terms of targeted individual or group counseling and/or treatment sessions are referred to as central for upholding mental wellbeing. Utilizing professionals with familiarity of policing culture is recommended as this can increase the chances of personnel seeking and receiving adequate help. Peer-counseling or peer support networks are mentioned alternatives to secure high attendance.	Burns et al. (2008), Burruss et al. 2018, Doyle et al. (2023), Fortune et al. (2018), Gewirtz-Meydan et al. (2023, 2024), Mitchell et al. (2023), Powell et al. (2014a), Redmond et al. (2023)
*Debriefing*	
Regular debriefing sessions or mental health check-ins are suggested as a way for personnel to support each other on a day-to-day basis. Reactions can also be discussed as part of regular staff meetings or peer walks, where personnel can counsel each other.	Burns et al. (2008), Gewirtz-Meydan et al. (2023, 2024), Mitchell et al. (2023), Wilson-Kovacs et al. (2022)
*Family support*	
Support from family and friends can provide an important buffer to the potential negative effects of working with CSA investigations. Involving family members in understanding the line of work and recognizing signs of hampered wellbeing can thus provide an extra safety net for online CSA personnel	Brady (2017), Perez et al. (2010), Seigfried-Spellar (2018)
*Wellness programs*	
Wellness program(s) are frequently suggested as an overarching measure to secure wellbeing among online CSA personnel. Although it may include other mentioned aspects, such as psychological support and training, it is usually a preexisting package provided by the organization or through consultants. Participation in mindfulness and emotional intelligence programs is also suggested in this literature.	Burruss et al. (2018), Gewirtz-Meydan et al. (2023), Leclerc et al. (2022), Mitchell et al. (2022, 2023)

*Note.* The suggestions presented are derived from both survey and interview studies. None of the suggestions are based on evaluations from interventions.

## Discussion

Our review on the wellbeing of online CSA police personnel included 33 articles, using both quantitative and qualitative methods for their analysis based on data from different national police forces. It should be noted, however, that several studies were based on data derived from the same population by the same authors, albeit focusing on different wellbeing outcomes or correlates (see Table 1). Given the nature of the work, it is perhaps not surprising that most studies had a focus on exposure, secondary trauma, and coping strategies. In terms of severity of symptoms, the findings from these studies indicate that there is a large variation in which police personnel are negatively affected by the continuous exposure to online CSA material. Consequently, although online CSA policing has the capacity to destructively influence both professional and personal lives (Leclerc et al., 2022), in many cases, the reported symptoms do not appear to exceed those found in the general population (Tehrani, 2016; Tomyn et al., 2015).

So far, explanations for whom are at risk of hampered wellbeing have mainly been sought by studying individual characteristics, such as gender (Gewirtz-Meydan et al., 2023) and personality (Tehrani, 2016), or relate these results to the use of different kinds of coping mechanisms (O’Brien et al., 2024). However, these characteristics only seem to explain a small proportion of the variance, with results being inconclusive between studies, or study design not controlling for other potential explanations. For example, the gender differences found in four studies could perhaps be explained by other factors, such as pressure to be more productive as a female police officer compared to male police officers to be accepted (Poikela et al., 2025). Hence, also more frequently exposed to stressors. Furthermore, as indicated in qualitative study results, the needs and experiences of online CSA police personnel are diverse, and therefore also need to be tackled across different levels, not only the individual but also on a group, leader/manager, and organizational and system level (Simonovska et al., 2023). Also, in the reviewed studies, the use of coping mechanisms is often assumed to be either beneficial or maladaptive. However, the results from the use of different strategies fluctuate. This could be seen as indicative of a non-static process in which the effectiveness of a certain strategy is influenced by several situational aspects. As suggested by Bonanno and Burton (2013), the flexibility in adapting one’s behavior may therefore also be seen as more important than the capacity to access a single coping strategy.

Thus, even though some individuals may be more sensitive or vulnerable because of, for example, their personality, even highly resilient individuals may experience impaired wellbeing in an unsupportive or adverse work environment. Therefore, it is of the essence to also consider the work environment as a whole (Demerouti & Bakker, 2025). The results of this review clearly reflect that levels of secondary traumatic experiences, as well as other wellbeing outcomes, coincide with several factors on different levels. For example, the use of coping strategies (Denk-Florea et al., 2020), level of team cohesion and managerial support (Simonovska et al., 2023), availability of organizational resources (e.g., staffing and training, Powell et al., 2014b), and advancement of technologies and updating of laws and regulations (Mitchell et al., 2022) appear to be highly important.

The measures proposed (see Table 4) reflect the multifaceted nature of the challenges and span across individual, interpersonal, organizational, and systemic levels. These include a range of interventions—from education on how to best cope with exposure (Gullon-Scott & Johnson, 2024) to building organizational processes and procedures (Mitchell et al., 2022), as well as a supportive culture (Redmond et al., 2023) to mitigate and counteract stress. It is important to note that the effectiveness of these suggestions has yet to be evaluated in this specific field of work.

Given that the reviewed studies are based on a limited number of police personnel in a few countries, the effectiveness of different interventions is also likely to depend on specific contextual factors. For example, the availability of professional psychological support may not be an option for some police forces but can be well-established in others. Therefore, in line with recommendations for designing complex multilevel interventions (de Lange et al., 2024), it is essential to consider both contextual suitability and individual needs when determining which specific measures are most appropriate (See Table 5).

**Table 5.** Critical Findings.

• Research on online CSA crime investigations and their effects on police personnel’s wellbeing is a recent but growing phenomenon, emerging as a result of the increased online CSA crime rate.
• Police personnel working with online CSA crime investigations are frequently exposed to potentially (secondary) traumatic events.
• Prevalence levels of secondary traumatic stress, as well as other indicators of hampered wellbeing, vary. Approximately 20 to 30% of the police personnel working with these investigations experience high levels; however, most are not severely affected.
• The use of different coping strategies when watching abusive material and to decompress afterwards can help prevent hampered wellbeing.
• Support from peers, the organization, family and friends, as well as policy and lawmakers, is often highlighted in interview studies as vital for recovery from traumatic experiences.
• Interventions on multiple levels to support the wellbeing of online CSA police personnel are suggested in most studies, but no evaluations to examine the efficiency of different measures have been presented so far.

### Implications

Our findings have several important implications for research, policy, and practice (see Table 6).

**Table 6.** Implications for Practice, Policy, and Research.

• Only police forces in a few countries have been studied so far. There is a need for a greater variety in studied populations to draw general conclusions across settings.
• Longitudinal and intervention studies are called for to understand more about effects over time of exposure to online CSA material and the use of coping strategies. As well as for identifying efficient and evidence-based methods to tackle mental ill-health in this context
• Policymakers and police management should allocate resources to enable speedy updates of laws and possibilities to access technological advancements. This to facilitate cross-national collaborations in crime investigations and help reduce the time spent reviewing online CSA material.
• Police organizations should develop adapted routines for monitoring and support of online CSA police personnels wellbeing. They should also consider educational and informative strategies to reduce stigmatization of this form of policing.
• Police schools could help future police personnel to become more familiar with this type of work by including training in online investigations and the technology used. They could also train students in identification of signs of a poor wellbeing, and ways to prevent and counteract mental ill-health.

The studies included in our review are based on a limited number of participating national police forces and are either cross-sectional survey studies or interview studies, of which several include only a few participants. Therefore, we suggest that future research should focus on increasing diversity by studying police forces from a multitude of countries. This can help increase the generalizability of findings and expose cultural differences between national police forces in how investigations of online CSA crimes are organized and how they cope with exposure to online CSA material.

There is also a lack of longitudinal and intervention evaluation studies. For example, we do not know if the coping strategies that are suggested (see Table 3) are beneficial over time. Using avoidant strategies, such as shutting off emotions, may increase short-term productivity but hamper wellbeing in the long run (Strickland et al., 2023). Similarly, although a lot of suggestions have been given on how to prevent and counteract hampered wellbeing (see Table 4), we know nothing of the effectiveness of these interventive recommendations. Ideally, such interventions should be co-designed with online CSA police personnel to ensure contextual relevance and alignment with practitioners’ lived experiences (Rantatalo et al., 2025). They should also be evaluated using rigorous designs such as randomized control trials to better inform on evidence-based best practices (Schelvis et al., 2015).

Beyond research, we recommend that policymakers and police management allocate resources—both nationally and internationally—for cooperation, knowledge sharing, and the updating of laws and regulations so that technologically advanced methods can be utilized effectively in investigations. Internet crimes are global in nature, and perpetrators quickly update their methods and their modus operandi for sharing CSA material online. To counter this, it is essential to foster international cooperation between police forces, invest in the development of new technologies to support the processing of online CSA material, and ensure rapid implementation and dissemination of up-to-date tools, including artificial intelligence. Falling behind in the adoption of cutting-edge technology remains a persistent challenge and a significant stressor for personnel in this field (Redmond et al., 2023), which could be reduced by further engagement from policymakers and police management.

Furthermore, police organizations, with the help of occupational health professionals, should develop tailored procedures and processes for monitoring and supporting the wellbeing of their personnel working with online CSA investigations. Considering the specific challenges of this line of work, incorporating routines for safeguarding the mental health of the personnel should be a priority. Similarly, routines for feedbacking results of investigations to all involved (Denk-Florea et al. 2020), as well as efforts to promote cross-functional teamwork (Simonovska et al., 2023), are also clearly expressed needs. Efforts to reduce the stigma surrounding this line of work should also be taken into consideration. For example, educating and informing other police departments on this line of investigations could help reduce the risk of contamination by association with the crime that has been observed as an additional stressor (Spencer et al., 2020).

Finally, we suggest that police education programs should include training in performing online crime investigations in general, and more specifically, online CSA crimes. Such training should also include information on how to cope with exposure to CSA material, how to identify signs of hampered wellbeing, and measures that can be taken to reduce and handle mental ill-health.

### Limitations

Although we adhered to the stepwise scoping review methodology to ensure a thorough analysis of the existing literature concerning the wellbeing of online CSA police personnel, there are several limitations that deserve attention. First, we limited our scope to peer-reviewed articles published in the English language. This may have excluded relevant findings published in other languages or as “gray” literature (e.g., reports or book chapters). Nearly all included articles are based on populations from English-speaking countries, which may indicate a culture-related selection bias.

Second, we only included literature with a clear reference to CSA personnel exposed to “online” material. This excludes studies with a CSA policing focus that does not involve exposure to digitalized material, even though the crime is the same and investigations may include examination of visual and/or auditory evidence. Widening the scope could thus have led to richer material to depart from in our analysis. Drawing this line can perhaps also be seen as arbitrary, given the crime-type communalities. However, choosing a narrower path also means that we do not extend conclusions beyond our scope.

Third, the results of our review revealed a substantial variation in the prevalence of indicators of hampered wellbeing among online CSA police personnel. These results are likely influenced by differences in the populations studied and their respective conditions, but may also be attributed to variations in research methods and the timing of the studies, as knowledge about coping strategies and preventive measures has evolved over time. Thus, given the limited number of studies available, determining the wellbeing state of the studied population remains a challenge. However, there seems to be a clear consensus on the risk for secondary trauma as a result of this line of work, and that more studies are needed that examine wellbeing and ways of mitigating the potential consequences of frequent exposure to online CSA material.

## Conclusions

In this scoping review, we aimed to synthesize the existing peer-reviewed articles on the wellbeing of police personnel working with online CSA investigations. Our findings show that the subject has received increased attention over the last decade. It also recognizes that this research is mainly based on a limited selection of national police forces and is mainly based on cross-sectional surveys and/or interview designs. Longitudinal and interventional research is missing. Our collated results present a comprehensive overview of findings on the wellbeing of this population. It also reveals how their wellbeing is associated with individual characteristics and use of coping strategies, as well as with demands and resources available in their workplaces. Furthermore, suggestions given for how wellbeing in this line of work can be promoted are summarized to inform and encourage future intervention efforts.

By summarizing available findings and gaps in knowledge on the challenges and opportunities for wellbeing in this field, we provide a foundation for future research, policy, and practice aimed at developing preventive strategies, evidence-based interventions, and healthy work practices. We urge stakeholders, including researchers, police organizations, law and policy makers, educators, and health professionals, to collectively engage in developing and disseminating ways of reducing and dealing with exposure to online CSA material. Ensuring the wellbeing of those on the frontline of online CSA investigations is not only an ethical imperative but a shared societal responsibility.

## Supplemental Material

sj-docx-1-tva-10.1177_15248380251358228 – Supplemental material for Wellbeing Among Police Personnel Investigating Online Child Sexual Abuse: A Scoping Review Supplemental material, sj-docx-1-tva-10.1177_15248380251358228 for Wellbeing Among Police Personnel Investigating Online Child Sexual Abuse: A Scoping Review by Robert Lundmark, Alva Lindholm, Ola Lindberg and Oscar Rantatalo in Trauma, Violence, & Abuse
