# Supplementary material for: Wellbeing Among Police Personnel Investigating Online Child Sexual Abuse: A Scoping Review
Source: Trauma Violence Abuse. 2025 Aug 20;27(4):1734–47. doi: 10.1177/15248380251358228 (PMC13578671; doi:10.1177/15248380251358228)
Supplement: Supplementary file 1 [file sj-docx-1-tva-10.1177_15248380251358228.docx]

**Appendix 1**

Data base search strings

***WoS***

Search 1:(online OR cyber* OR digital OR internet ) NEAR/5 (sex* OR grooming OR abuse OR pornography OR "exploitation material" ) NEAR/5 (child* OR minor* OR infant* OR adolescent* ) (Topic) and police OR policing OR "law enforcement*" OR "crime investigation*" OR "criminal investigation*" (Topic) and Article or Review Article (Document Types) and 2024 or 2023 or 2022 or 2021 or 2020 or 2019 or 2018 or 2017 or 2016 or 2015 or 2014 or 2013 or 2012 or 2011 or 2010 or 2009 or 2008 or 2007 or 2006 or 2005 or 2004 or 2001 (Publication Years) and English (Languages)

Search 2:police OR policing OR "law enforcement*" OR "crime investigation*" OR "criminal investigation*" (Topic) and "child sexual abuse and exploitation" OR csae OR csec OR "commercially sexually exploited children" OR "online child sexual exploitation and abuse" OR "online child sexual abuse" OR "internet crimes against children" OR "child sexual abuse material" OR grooming OR "child abuse material" OR "child exploitation material" OR "child pornography" OR csam OR csea OR "online child sexual exploitation" OR "child sexual exploitation material" OR "child sexual exploitation and abuse" (Topic) and Article or Review Article (Document Types) and 2024 or 2023 or 2022 or 2021 or 2020 or 2019 or 2018 or 2017 or 2016 or 2015 or 2014 or 2013 or 2012 or 2011 or 2010 or 2009 or 2008 or 2007 or 2006 or 2005 or 2004 or 2002 (Publication Years) and English (Languages)

Search 3: #1 OR #2

***Scopus***

Search 1: ( TITLE-ABS-KEY ( ( online OR cyber* OR digital OR internet ) W/5 ( sex* OR grooming OR abuse OR pornograph* OR "exploitation material" ) W/5 ( child* OR minor* OR infant* OR adolescent* ) ) AND TITLE-ABS-KEY ( police OR policing OR "law enforcement*" OR "crime investigation*" OR "criminal investigation*" ) ) AND PUBYEAR > 1999 AND PUBYEAR < 2025 AND ( LIMIT-TO ( DOCTYPE , "ar" ) OR LIMIT-TO ( DOCTYPE , "re" ) ) AND ( LIMIT-TO ( LANGUAGE , "English" ) ) AND ( LIMIT-TO ( SRCTYPE , "j" ) )

Search 2: ( TITLE-ABS-KEY ( "child sexual abuse and exploitation" OR csae OR csec OR "commercially sexually exploited children" OR "online child sexual exploitation and abuse" OR "online child sexual abuse" OR "internet crimes against children" OR "child sexual abuse material" OR grooming OR "child abuse material" OR "child exploitation material" OR "child pornography" OR csam OR csea OR "online child sexual exploitation" OR "child sexual exploitation material" OR "child sexual exploitation and abuse" ) AND TITLE-ABS-KEY ( police OR policing OR "law enforcement*" OR "crime investigation*" OR "criminal investigation*" ) ) AND PUBYEAR > 1999 AND PUBYEAR < 2025 AND ( LIMIT-TO ( DOCTYPE , "ar" ) OR LIMIT-TO ( DOCTYPE , "re" ) ) AND ( LIMIT-TO ( SRCTYPE , "j" ) ) AND ( LIMIT-TO ( LANGUAGE , "English" ) )

Search 3: #1 OR #2

***SOC index***

Search 1:DE "CHILD sexual abuse investigation" OR TI "CHILD sexual abuse investigation" OR AB "CHILD sexual abuse investigation" OR KW "CHILD sexual abuse investigation"

Search 2: DE ("CRIMINAL investigation") OR DE ("POLICE")) OR DE ("LAW enforcement") ) OR TI ( "criminal investigation*" OR "crime investigation*" OR police OR policing OR "law enforcement" ) OR AB ( "criminal investigation*" OR "crime investigation*" OR police OR policing OR "law enforcement" ) OR KW ( "criminal investigation*" OR "crime investigation*" OR police OR policing OR "law enforcement" )

Search 3: DE "CHILD sexual abuse" OR TI "CHILD sexual abuse" OR AB "CHILD sexual abuse" OR KW "CHILD sexual abuse"

Search 4: DE "INTERNET" OR TI ( internet OR cyber* OR online OR digital ) OR AB ( internet OR cyber* OR online OR digital ) OR KW ( internet OR cyber* OR online OR digital )

Search 5: S2 AND S3 AND S4

Search 6: SU ( "child sexual abuse and exploitation" OR csae OR csec OR "commercially sexually exploited children" OR "online child sexual exploitation and abuse" OR "online child sexual abuse" OR "internet crimes against children" OR "child sexual abuse material" OR grooming OR "child abuse material" OR "child exploitation material" OR "child pornography" OR csam OR csea OR "online child sexual exploitation" OR "child sexual exploitation material" OR "child sexual exploitation and abuse" ) OR TI ( "child sexual abuse and exploitation" OR csae OR csec OR "commercially sexually exploited children" OR "online child sexual exploitation and abuse" OR "online child sexual abuse" OR "internet crimes against children" OR "child sexual abuse material" OR grooming OR "child abuse material" OR "child exploitation material" OR "child pornography" OR csam OR csea OR "online child sexual exploitation" OR "child sexual exploitation material" OR "child sexual exploitation and abuse" ) OR AB ( "child sexual abuse and exploitation" OR csae OR csec OR "commercially sexually exploited children" OR "online child sexual exploitation and abuse" OR "online child sexual abuse" OR "internet crimes against children" OR "child sexual abuse material" OR grooming OR "child abuse material" OR "child exploitation material" OR "child pornography" OR csam OR csea OR "online child sexual exploitation" OR "child sexual exploitation material" OR "child sexual exploitation and abuse" ) OR KW ( "child sexual abuse and exploitation" OR csae OR csec OR "commercially sexually exploited children" OR "online child sexual exploitation and abuse" OR "online child sexual abuse" OR "internet crimes against children" OR "child sexual abuse material" OR grooming OR "child abuse material" OR "child exploitation material" OR "child pornography" OR csam OR csea OR "online child sexual exploitation" OR "child sexual exploitation material" OR "child sexual exploitation and abuse")

Search 7: S2 AND S6

Search 8: SU ( ( online OR cyber* OR digital OR internet ) N5 ( sex* OR grooming OR abuse OR pornograph* OR "exploitation material" ) N5 ( child* OR minor* OR infant* OR adolescent* ) ) OR AB ( ( online OR cyber* OR digital OR internet ) N5 ( sex* OR grooming OR abuse OR pornograph* OR "exploitation material" ) N5 ( child* OR minor* OR infant* OR adolescent* ) ) OR TI ( ( online OR cyber* OR digital OR internet ) N5 ( sex* OR grooming OR abuse OR pornograph* OR "exploitation material" ) N5 ( child* OR minor* OR infant* OR adolescent* ) ) OR KW ( ( online OR cyber* OR digital OR internet ) N5 ( sex* OR grooming OR abuse OR pornograph* OR "exploitation material" ) N5 ( child* OR minor* OR infant* OR adolescent* ) )

Search 9: S2 AND S8

Search10: S1 OR S5 OR S7 OR S9 (Peer reviewd, date2000-2024, English)

***APA Psycinfo***

Search 1 : DE "Law Enforcement" OR TI ( "criminal investigation*" OR "crime investigation*" OR police OR policing OR "law enforcement" ) OR AB ( "criminal investigation*" OR "crime investigation*" OR police OR policing OR "law enforcement" ) OR KW ( "criminal investigation*" OR "crime investigation*" OR police OR policing OR "law enforcement" )

Search 2: DE "Child Sexual Abuse" OR TI "Child Sexual Abuse" OR AB "Child Sexual Abuse" OR KW "Child Sexual Abuse"

Search 3: DE "Internet" OR TI ( digital OR cyber OR online OR internet ) OR AB ( digital OR cyber OR online OR internet ) OR KW ( digital OR cyber OR online OR internet )

Search 4: #2 AND 3

Search 5: #1 AND #4

Search 6: DE "Sexting" OR TI sexting OR AB sexting OR KW sexting

Search 7: SU ( child* OR adolescent* OR minor* OR infant* ) OR AB ( child* OR adolescent* OR minor* OR infant* ) OR KW ( child* OR adolescent* OR minor* OR infant* ) OR TI ( child* OR adolescent* OR minor* OR infant* )

Search 8: #6 AND #7

Search 9: #1 AND #8

Search 10: SU ( "child sexual abuse and exploitation" OR csae OR csec OR "commercially sexually exploited children" OR "online child sexual exploitation and abuse" OR "online child sexual abuse" OR "internet crimes against children" OR "child sexual abuse material" OR grooming OR "child abuse material" OR "child exploitation material" OR "child pornography" OR csam OR csea OR "online child sexual exploitation" OR "child sexual exploitation material" OR "child sexual exploitation and abuse" ) OR TI ( "child sexual abuse and exploitation" OR csae OR csec OR "commercially sexually exploited children" OR "online child sexual exploitation and abuse" OR "online child sexual abuse" OR "internet crimes against children" OR "child sexual abuse material" OR grooming OR "child abuse material" OR "child exploitation material" OR "child pornography" OR csam OR csea OR "online child sexual exploitation" OR "child sexual exploitation material" OR "child sexual exploitation and abuse" ) OR AB ( "child sexual abuse and exploitation" OR csae OR csec OR "commercially sexually exploited children" OR "online child sexual exploitation and abuse" OR "online child sexual abuse" OR "internet crimes against children" OR "child sexual abuse material" OR grooming OR "child abuse material" OR "child exploitation material" OR "child pornography" OR csam OR csea OR "online child sexual exploitation" OR "child sexual exploitation material" OR "child sexual exploitation and abuse" ) OR KW ( "child sexual abuse and exploitation" OR csae OR csec OR "commercially sexually exploited children" OR "online child sexual exploitation and abuse" OR "online child sexual abuse" OR "internet crimes against children" OR "child sexual abuse material" OR grooming OR "child abuse material" OR "child exploitation material" OR "child pornography" OR csam OR csea OR "online child sexual exploitation" OR "child sexual exploitation material" OR "child sexual exploitation and abuse")

Search 11: #1 AND #10

Search 12: SU ( ( online OR cyber* OR digital OR internet ) N5 ( sex* OR grooming OR abuse OR pornograph* OR "exploitation material" ) N5 ( child* OR minor* OR infant* OR adolescent* ) ) OR AB ( ( online OR cyber* OR digital OR internet ) N5 ( sex* OR grooming OR abuse OR pornograph* OR "exploitation material" ) N5 ( child* OR minor* OR infant* OR adolescent* ) ) OR TI ( ( online OR cyber* OR digital OR internet ) N5 ( sex* OR grooming OR abuse OR pornograph* OR "exploitation material" ) N5 ( child* OR minor* OR infant* OR adolescent* ) ) OR KW ( ( online OR cyber* OR digital OR internet ) N5 ( sex* OR grooming OR abuse OR pornograph* OR "exploitation material" ) N5 ( child* OR minor* OR infant* OR adolescent* ) )

Search 13: #1 AND #12

Search 14: #5 OR #9 OR #11 OR #13

Limiters Scholarly (Peer Reviewed) Journals; Publication Year: 2000-2024 Language English
